# Terahertz Encryption With a Polarization‐Sensitive Metasurface

**DOI:** 10.1002/nap2.70123

**Published:** 2026-05-06

**Authors:** Yixuan He, Huan Zhao, Tong Nan, Xinke Wang, Yan Zhang

**Affiliations:** ^1^ Department of Physics Capital Normal University Beijing China; ^2^ School of Optics and Photonics Beijing Institute of Technology Beijing China

**Keywords:** encryption, metasurface, polarization‐sensitive, terahertz

## Abstract

The rapid advancement of information age has created an urgent need for integrated physical‐layer security solutions. We propose a dual‐functional terahertz (THz) encryption metasurface that enables independent information retrieval in both the near field and the far field within a single device. By precisely tailoring the complex amplitude of orthogonal linear polarization components, the metasurface forms two spatially separated far‐field channels for left‐ and right‐handed circular polarizations, which can be accessed simultaneously under linearly polarized illumination and are theoretically free from interchannel crosstalk. In the near field, polarization‐selective amplitude modulation reveals textual patterns, whereas in the far field, phase engineering encodes multiple digital data in focal‐spot arrays under linear, left‐handed, and right‐handed circular polarization states. This compact metasurface provides a scalable route toward THz security tags and robust physical‐layer protection for identification, anticounterfeiting, and high‐capacity wireless communications.

## Graphical abstract

We propose a dual‐functional terahertz encryption metasurface that enables independent information retrieval in both the near field and the far field within a single device. In the near field, polarization‐selective amplitude modulation reveals textual patterns, whereas in the far field, phase engineering encodes multiple digital data in focal‐spot arrays under linear, left‐handed, and right‐handed circular polarization states.

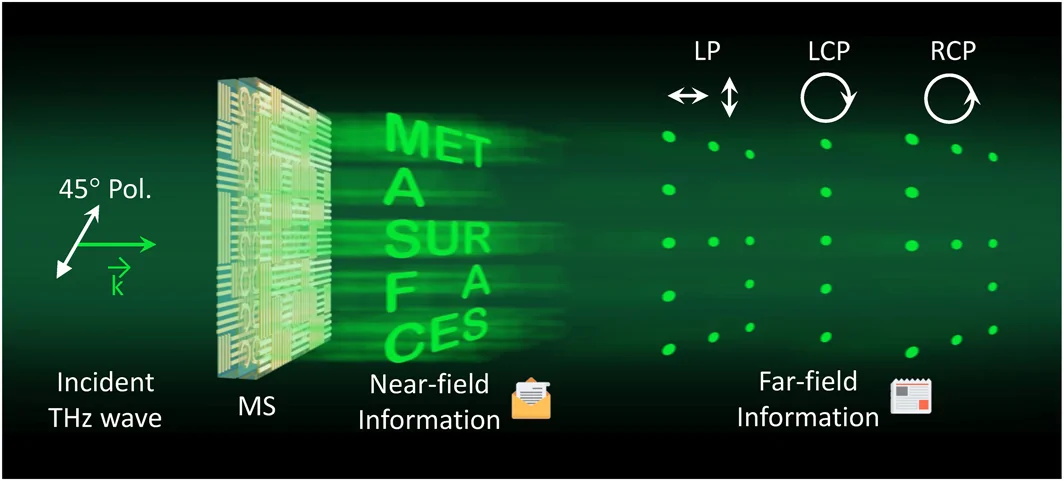

## 1 Introduction

In the information age, the explosive growth of data transmission and storage has made information security a central technological challenge. Optical encryption has emerged as a powerful strategy owing to the intrinsic parallelism, high bandwidth, and multidimensional degrees of freedom of light [1, 2, 3, 4], making it particularly suitable for applications such as watermarking and anticounterfeiting [5, 6, 7]. The existing optical encryption systems include double random phase encoding (DRPE) system [8], off‐axis holography system [9], ghost imaging system [10], and ptychography system [11]. They are bulky and usually has limited controlled physical dimensions in both encrypted channel and key space due to their unsufficient ablities in multidimensional modulation for electromagnetic wave. Meanwhile, emerging data‐driven methods, including deep‐learning‐ and neural‐network‐assisted information processing, have also opened new possibilities for high‐capacity optical information encoding and encryption [12, 13].

Metasurfaces, as two‐dimensional arrays of engineered subwavelength structures, have emerged as a versatile platform for offering unprecedented control over electromagnetic waves based on its flexible modulated abilities of amplitude, phase, and polarization state [14, 15, 16, 17]. This capability has driven significant research into metasurface‐based devices, leading to various devices for wavefront manipulation, including metalenses [18, 19, 20, 21], beam shapers [22, 23, 24, 25], and special beam generators [26, 27, 28, 29]. Beyond these wavefront‐control applications, metasurfaces have also been explored for information encryption, leveraging various physical dimensions as encryption keys. These dimensions include wavelength [30], spatial position [31, 32, 33], optical angular momentum [34, 35, 36], and polarization state [37, 38, 39] as well as their combinations [40, 41, 42]. Among these, the polarization state of light holds exceptional promise for encryption devices, owing to the vast information capacity and the existence of mutually orthogonal eigenstates, such as orthogonal linearly polarized (LP) states or left‐ and right‐handed circularly polarized (LCP and RCP) states.

The polarization‐sensitive metasurfaces has been developed from utilizing basic polarization channels to integrating multiple degrees of freedom, significantly enhancing the encryption security and functional capacity. The most common method for polarization‐sensitive encryption devices relies on the polarization‐sensitive response of metasurfaces. For instance, leveraging different Pancharatnam–Berry (PB) phase responses to LCP and RCP wave enables at least two‐channel image hiding using a single metasurface [37]. Furthermore, by applying different phase modulation on *x*‐ and *y*‐polarized waves, different images can be concealed in their respective polarization channels [43]. Moreover, by combining the PB phase with the propagation phase, one can further extend the polarization multiplexing capability and achieve independent wavefront control of LP as well as LCP and RCP wave [44, 45, 46, 47, 48, 49]. Building on this idea, Liu et al. demonstrated a transmission‐mode all‐dielectric metasurface that fully decouples arbitrary amplitude and phase profiles into two orthogonal polarization states by jointly exploiting PB and propagation phases, enabling polarization‐switchable near‐field nanoprinting and far‐field complex‐amplitude holography within a single metasurface [49]. They further showed chirality‐selective operation under LCP/RCP wave, where distinct nanoprinted patterns and holographic images can be retrieved by switching the incident handedness. More recently, Ning et al. has demonstrated polarization‐sensitive encryption via a metasurface‐enabled visual secret sharing scheme, where global optimization across the entire metasurface realizes eight independent polarization channels with minimized crosstalk [50]. Related studies on spin‐dependent light manipulation, such as the photonic spin Hall effect on metasurfaces, have also provided further physical insight into polarization‐dependent wavefront control [51]. Despite these advances, PB phase‐based multiplexing of LCP and RCP channels typically relies on polarization‐dependent illumination and analyzer‐assisted readout. When the incident field contains mixed spin components, the intrinsically opposite‐sign PB phase responses for LCP and RCP yield coexisting spin‐converted fields that may superpose, resulting in undesired background if the channels are not properly isolated.

Compared with visible and infrared optical encryption, terahertz (THz) encryption offers a distinct application space owing to the unique propagation characteristics of THz waves. In particular, THz radiation can penetrate a broad range of materials that are opaque at visible wavelengths but nonconductive to terahertz waves, such as paper, plastics, textiles, and ceramics, making it attractive for concealed security tags, through‐package authentication, and anticounterfeiting applications. In addition, the THz band is also of interest for short‐range high‐capacity wireless links, where physical‐layer encryption implemented directly at the electromagnetic wavefront can serve as a useful complement to conventional digital cryptographic approaches. At the same time, the longer wavelength of THz radiation leads to a comparatively lower spatial resolution than that in visible and infrared systems, which should be taken into account in the design of practical THz encryption devices. These features make THz metasurfaces particularly promising for secure identification and communication applications [52, 53, 54, 55, 56].

Addressing these challenges, we propose a dual‐functional THz encryption metasurface that concurrently operates in the near‐ and far‐fields. The device leverages independent control over the amplitude and phase of orthogonal polarizations to construct decoupled far‐field LCP and RCP channels. Benefiting from the metasurface's accurate and independent amplitude–phase control of the *x*‐ and *y*‐polarized components, the resulting far‐field LCP and RCP responses are free of interchannel crosstalk, theoretically. Importantly, both channels can be accessed simultaneously under a single linearly polarized illumination, eliminating the need for sequential polarization switching of the light source. Notably, the metasurface simultaneously supports dual‐modes encryption, enabling independent information retrieval in the near field and far field. Textual patterns are revealed in the near field via polarization‐selective amplitude modulation. Simultaneously, in the far field, phase manipulation encodes distinct digital data streams, projecting them as focal spot arrays for LP, LCP, and RCP states. This work establishes a compact near‐/far‐field integrated physical‐layer encryption paradigm, offering scalable polarization‐multiplexed metasurface platform for identification, anticounterfeiting, and high‐capacity wireless communication systems.

## 2 Design

The working principle of the proposed encryption device is shown in Figure 1. As illustrated in Figure 1a, the metasurface functions as a multilevel information encryption device with a dual‐mode data encoding function. The device directly displays the text “METASURFACES” in the near‐field region. Although probed in the far‐field, the same metasurface encodes polarization state selected digital information composed of focal spot arrays. Specifically, distinct numbers “6”, “1”, and “5” are decoded under LP, LCP, and RCP detection. This dual‐mode operation allows the same device to function as both a readable data storage medium and a reconfigurable information platform. This polarization‐encoded encoding mechanism ensures that only authorized user possessing the precise polarization key can successfully decode the information.

**FIGURE 1:**
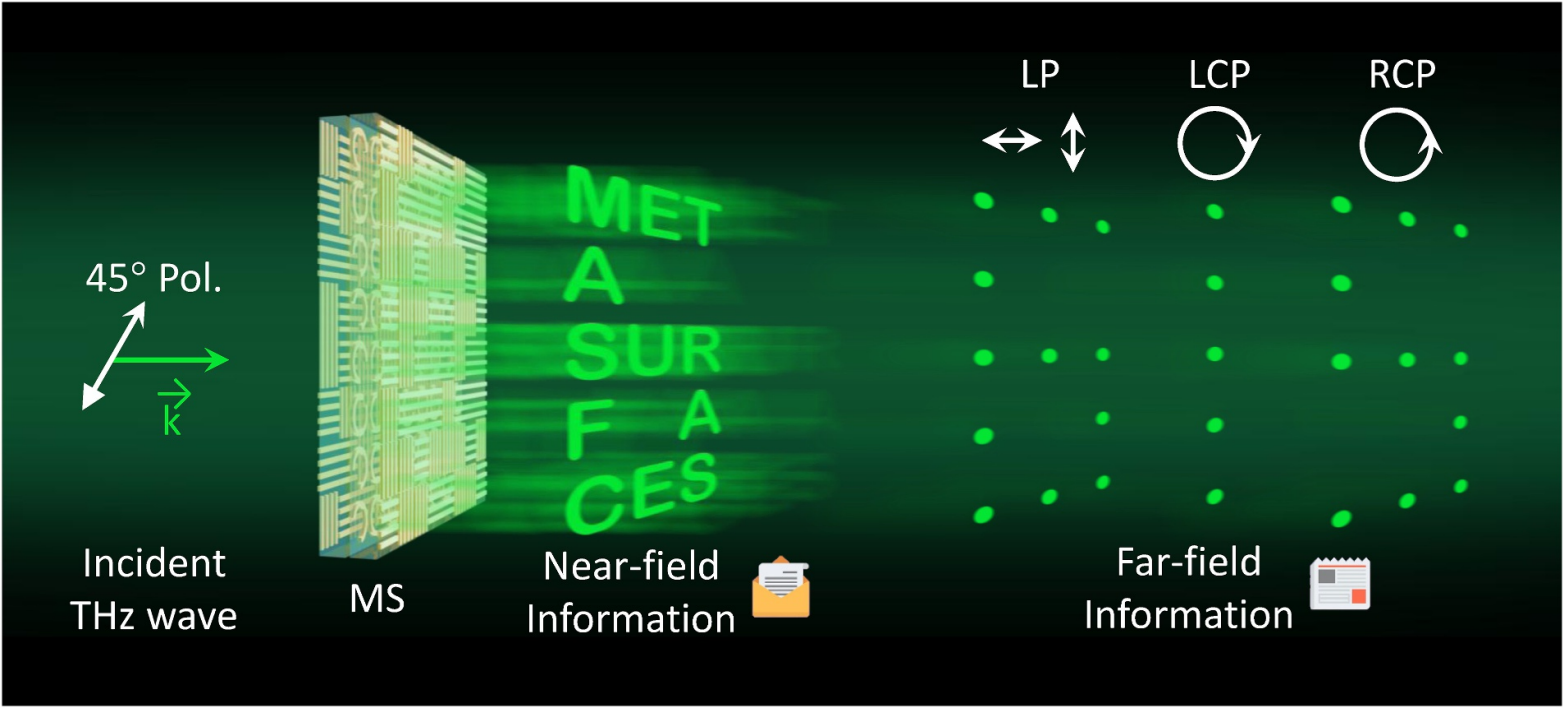
Schematic of the dual‐functional terahertz encryption device. The near‐field pattern spells the word “METASURFACES”, serving as a verifiable fingerprint. In the far field, polarization‐dependent digital data (e.g., “202”, “503”, and “248”) are encoded as distinct focal spot arrays under linear (LP), left‐ (LCP), and right‐circular (RCP) polarization states, enabling multichannel data transmission.

The design flow of the dual‐functional THz encryption metasurface device is illustrated in Figure 2a. The first step involves visualization of the near‐ and far‐field information to be encoded. Taking the designed sample #S1 as an example, this sample encodes the word “METASURFACES” in the near‐field, whereas under LP, LCP, and RCP detection, it encodes the numbers 6, 1, and 5 in the far‐field, respectively. Specifically, the far‐field information of the numbers is represented by a 5 × 3 array, the near‐field information of the word is arranged within the morphology of the number in LP state.

**FIGURE 2:**
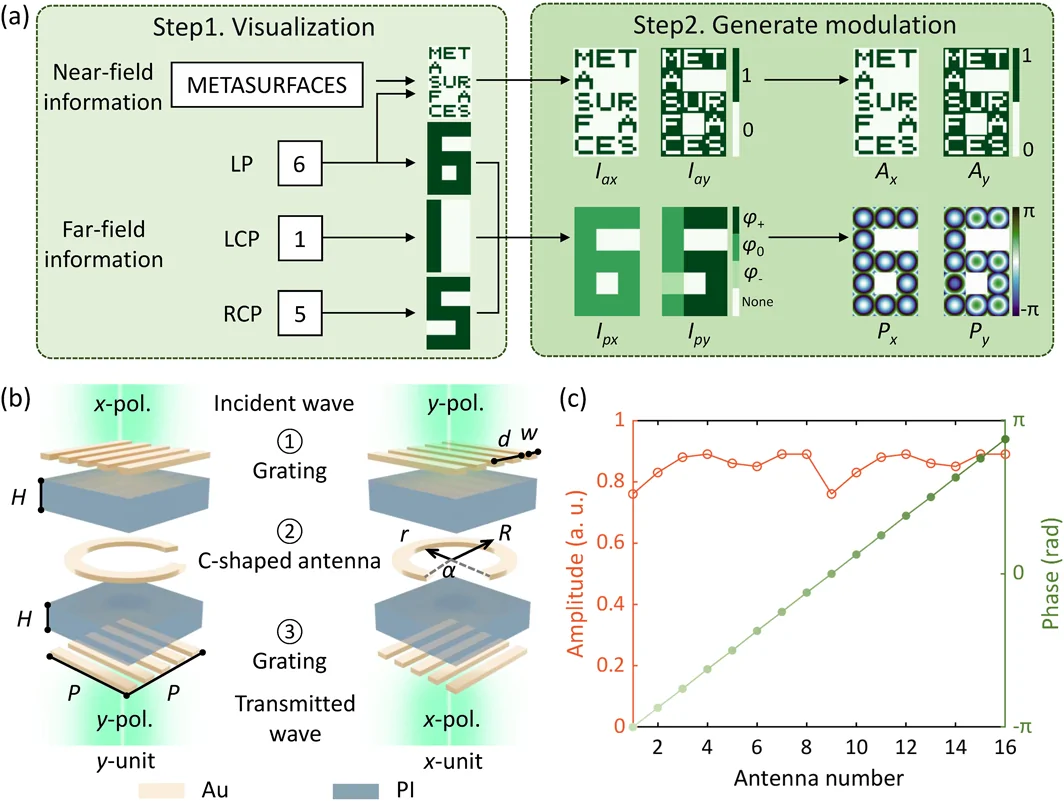
Design principle of dual‐functional THz metasurface. (a) Design flowchart of principle of dual‐functional THz encryption device. (b) Schematic of the designed metasurface. *p* = 100 μm, *H* = 40 μm, *w* = 10 μm, *d* = 20 μm, *R* = 45 μm, and *r* = 35 μm. *α* is the opening angle of the C‐shaped antenna. (c) The amplitude transmission and phase modulation of the selected 16 *y*‐units, the red‐circles and line denote the amplitude transmission, changing in the range of 0.7–0.9, whereas the green‐dots and line denote the phase modulation, covering the range of ‐π to π.

The second step is to generate the required amplitude and phase modulations of the transmitted *x*‐ and *y*‐polarized waves for the metasurface. In this step, we first create the amplitude and phase index matrices for the *x*‐ and *y*‐polarizations. The near‐field information controls the amplitude index matrices, *I*_*ax*_ and *I*_*ay*_ denote the amplitude index matrices for the transmitted *x*‐ and *y*‐polarized waves, respectively. It can be seen that the amplitude index matrices for *x*‐ and *y*‐polarization displays the positive and the negative pattern of the word, respectively. The opposite values of the two matrices represent spatial multiplexing of the *x*‐ and *y*‐polarized wave. From these matrices, the relative amplitude modulations for the *x*‐ and *y*‐polarized waves are obtained, taking values of either 0 or 1. The phase modulation of the transmitted wave is the key to achieving polarization‐sensitive far‐field information encoding. A lens phase is assigned to each position of the 5 × 3 array. Noted that LCP and RCP wave can be expressed as combinations of the electric field components *E*_*x*_ and *E*_*y*_ as follows:

(1) $$\begin{array}{c} E_{\text{LCP}}=1/\sqrt{2}\left(E_{x}+iE_{y}\right)\\ E_{\text{RCP}}=1/\sqrt{2}\left(E_{x}-iE_{y}\right). \end{array}$$

To obtain distinct foci‐array displays under LCP and RCP detection, we construct three different lens phases, $\varphi_{0},\varphi_{-}\;\text{and}\;\varphi_{+}$, which has a ‐π/2 and π/2 phase shift relative to *φ*_*0*_, respectively. Their expressions are given below:

(2) $$\begin{array}{ll} \varphi_{0}\left(x,y\right) & =\frac{2\pi }{\lambda }\left(f-\sqrt{x^{2}+y^{2}+f^{2}}\right)\\ \varphi_{-}\left(x,y\right) & =\varphi_{0}\left(x,y\right)-\pi /2\\ \varphi_{+}\left(x,y\right) & =\varphi_{0}\left(x,y\right)+\pi /2, \end{array},$$

where *λ* is the incident wavelength, which is 400 μm, and *f* is the focal length of the metalens, which is 4 mm in this design. *I*_*px*_ and *I*_*px*_ represent the phase index matrices for the transmitted *x*‐ and *y*‐polarized waves and are composed of $\varphi_{0},\varphi_{-}\;\text{and}\;\varphi_{+}$. These different phase distributions of lenses will generate different foci‐array distribution for LP, LCP, and RCP waves. Then, by arranging the corresponding lens phases according to the phase index matrices, we can obtain the phase modulation for the transmitted *x*‐ and *y*‐polarized waves.

We have designed two types of metasurface units, which are *y*‐ and *x*‐unit offering high transmission and phase modulation for transmitted *y*‐ and *x*‐polarized wave, respectively. The designed metasurface is shown in Figure 2b. The top and bottom metallic layers (① & ③) are orthogonal grating arrays served as polarized filter to make sure the polarization state of the transmitted wave is pure LP. The azimuth of the grating in layers ①/③ are 90°/0° and 0°/90° for the *y*‐ and *x*‐unit, respectively. The middle metallic layer (②) is composed of C‐shaped‐split‐ring antenna, which offers phase modulations for the transmitted cross‐polarized wave. The opening angle of C‐shaped antenna is α which can be tuned to change the phase of the transmitted cross‐polarized wave. The tri‐layer metallic structure converts the transmitted wave to a cross‐polarized state relative to the incident wave, while providing full 2π phase modulation for the cross‐polarized transmitted component. The two space layers are made of polyimide (PI) and have the same thickness. All these layers generate a Fabry–Perot‐like resonant cavity to achieve a high operating efficiency in transmission from 70% to 90%. The Jones matrix of the designed metasurface can be expressed as follows:

(3) $$J_{\left(x,y\right)}=\left[\begin{array}{cc} \frac{1}{2}\sin\;2\theta & -\cos^{2}\;\theta \\ \sin^{2}\;\theta & -\frac{1}{2}\sin\;2\theta \end{array}\right]\cdot t_{cross}\;\exp\left[i\varphi_{cross\left(x,y\right)}\left(\alpha \right)\right],$$

where (*x*, *y*) is arbitrary plane coordinate, *θ* is the polarized angle of the transmitted wave, which are 90° and 0° for the *y*‐ and *x*‐unit, respectively. The *t*_cross_ is the amplitude transmission of the transmitted cross‐polarized wave. The *φ*_cross_ is phase modulation of the transmitted cross‐polarized wave, which is a function of the opening angle α of the C‐shaped antenna.

The commercial software *FDTD Solutions* has been employed to build the model and to optimize the parameters of the metasurface unit (See details in Part 1 of SI). The working frequency is set as 0.75 THz. The thickness of the PI layers *H* and the three metallic layers are 40 μm and 150 nm, respectively. The period of unit *P* is optimized as 100 μm. The width and period of the metagratings *w* and *d* are 10 and 20 μm, respectively. The outer and inner radius of the C‐shaped antenna are *r* and *R* which are optimized as 35 and 45 μm, respectively. We have chosen 16 *y*‐units and 16 *x*‐units to modulate the phase of transmitted *y*‐ and *x*‐ polarized wave, respectively. The simulated amplitude and phase modulation of 16 *y*‐units are shown in Figure 2c. One can see the amplitude changes in the range of 0.7–0.9 whereas the phase is changing in the range of [‐π, π) with a step of π/8. Similarly, using the selected 16 *x*‐units can achieve 2π phase modulation and high amplitude transmission for *x*‐polarized wave. The simulated and measured transmission amplitudes of the Jones‐matrix elements for representative *y*‐unit #1 and *x*‐unit #1 are shown in Supporting Information S1: Figure S1 in the SI, confirming the polarization‐dependent cross‐polarized transmission behavior of the designed metasurface units. The result demonstrate that the device is broadband and highly efficient. The detailed geometric parameters of all these 32 units can be found in Supporting Information S1: Figure S2 in SI.

## 3 Results and Discussion

Based on the aforementioned design principles, we have designed three metasurface encryption devices encoding distinct information, which are labeled #S1, #S2, and #S3. All devices operate at a frequency of 0.75 THz, with a far field observation distance (i.e., focal length of lens) of 4 mm and a device size of 9 mm × 15 mm.

The amplitude modulations of these devices are shown in Figure 3a. It can be observed that the three devices encode the words “METASURFACES”, “MULTI‐CHANNEL”, and “IMAGE‐HIDING” in the near field, respectively. The modulation patterns for *x* and *y* polarizations are complementary and convey the same word information. The phase modulations are presented in Figure 3b. All lenses for *x*‐polarization consist of *φ*_*0*_, whereas the lenses for *y*‐polarization are assigned as $\varphi_{0},\varphi_{-},\text{or}\;\varphi_{+}$ at predesigned specific positions. The far‐field intensity distributions at *z* = 4 mm were simulated using Fresnel diffraction algorithm (See Part 2 in SI for details), and the results are shown in Figure 3c. Under *y*‐polarized detection, the intensity distribution of #S1, #S2, and #S3 exhibit focal arrays forming the numbers “6”, “8”, and “9”, respectively, matching their phase modulation patterns. Moreover, under LCP detection, the intensity distributions show the numbers “1”, “0”, and “3”, whereas under RCP detection, they display the numbers “5”, “2”, and “4”. These results are consistent with the +π/2 or −π/2 phase differences introduced at corresponding positions in the metasurface phase modulation.

**FIGURE 3:**
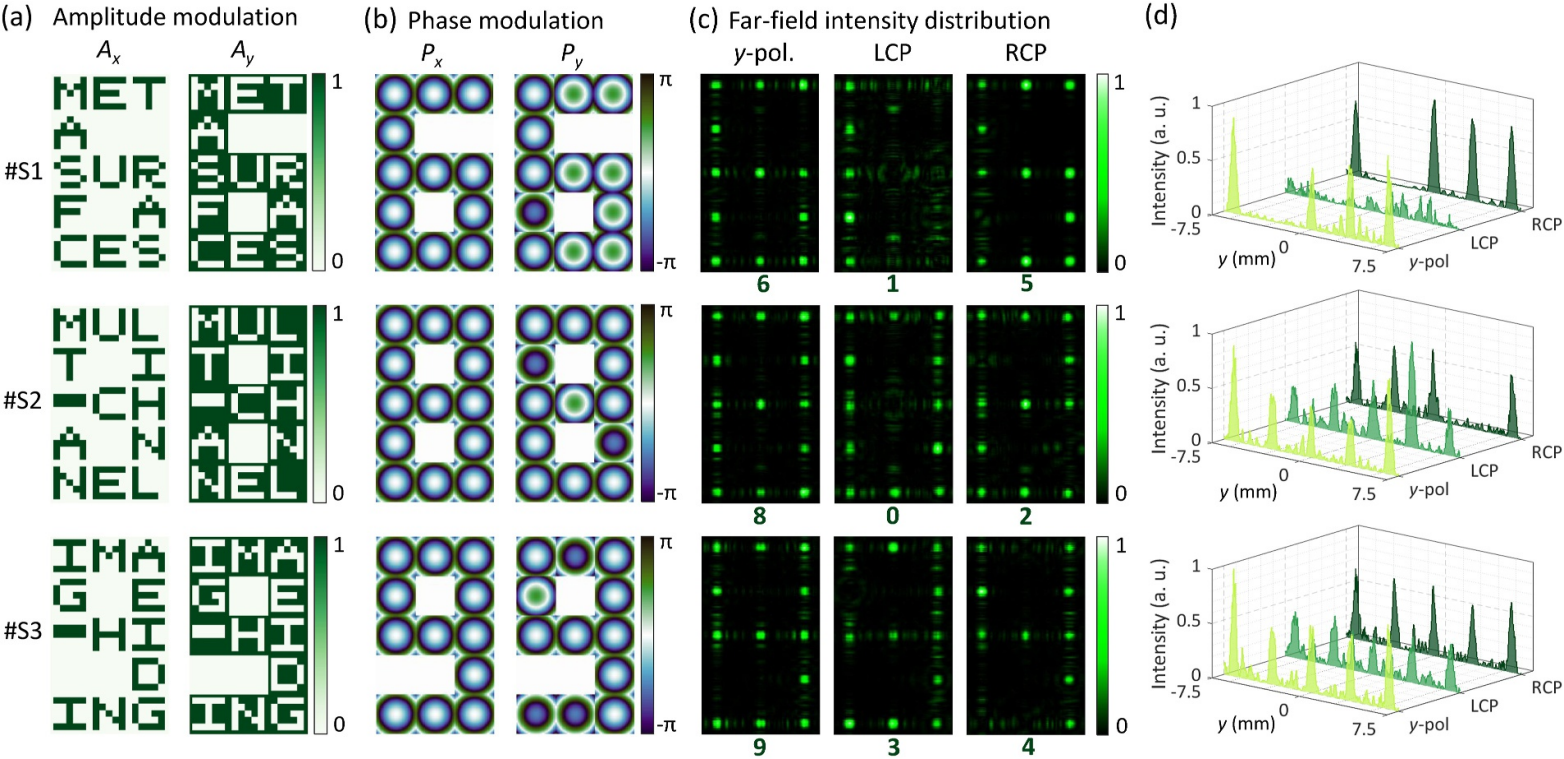
Characterization of dual‐functional metasurface encryption devices. (a) Amplitude modulations of #S1, #S2, and #S3. (b) Phase modulations of #S1, #S2, and #S3. (c) Simulated far‐field intensity distributions at the focal plane (*z* = 4 mm) under *y*‐polarized (top row), LCP (middle row), and RCP (bottom row) detection. The focal arrays correspond to numerals “6, 8, 9” under *y*‐polarization, “1, 0, 3” under LCP, and “5, 2, 4” under RCP for #S1, #S2, and #S3, respectively. (d) Cross‐sectional intensity profiles along the *y*‐axis at *x* = 7.515 mm, extracted from the intensity distributions in (c). Peaks (intensity between 0.5–0.8) indicate focal positions, whereas off‐focus regions maintain average intensities below 0.2.

To quantify the focal performance, intensity profiles along the *y*‐axis at *x* = 7.515 mm are extracted from these intensity distributions as shown in Figure 3d. The corresponding detection polarizations are indicated in the figure. It is confirmed that at positions where no focus is expected, the average intensity remains below 0.2, whereas at positions where has focal points, the intensity varies between 0.5 and 0.8. The nonuniform intensity distribution around focal spots arise from the different letter‐pattern‐shaped amplitude modulations applied to each lens element.

We have fabricated all these devices by conventional UV lithography, thermal evaporation, and lift‐off techniques (See Part 3 in SI for details). The microscope images of structure in the top, middle, and bottom layers are shown in Figure 4a, one can see the metagrating and the C‐shaped antennas are fabricated well. A home‐made THz focal plane imaging system which can detect the complex amplitude distribution of THz wave is used to measure the performances of fabricated devices. A schematic of the experimental setup is shown in Figure 4b. To measure both near‐field and far‐field information, two distinct detection planes are established. The near‐field detection plane (*P*_*n*_) is positioned at a distance of *D*_*n*_ = 400 μm from the samples. The far‐field detection plane (*P*_f_) is set at the focal length (*f* = 4 mm) of the designed focusing lens. A Ti:sapphire regenerative amplifier is used to produce a 100‐fs ultrashort laser pulse with a central wavelength of 800 nm and a repetition rate of 1 kHz. The laser beam with an average power of 2.3 W is divided into two parts, which are then used as the pump (2 W) and probe (300 mW) beams for the generation and detection of the THz waves, respectively. In the THz generation path, the pump beam impinged on a 1‐mm‐thick 〈110〉 ZnTe crystal to generate the THz radiation via optical rectification. A THz half‐wave plate (THWP) and a THz polarizer (TP) are used to produce and maintain a 45°‐linearly polarized incident THz wave. The transmitted THz wave is detected using another 〈110〉 ZnTe crystal. In the probe path, a combination of a half‐wave plate (HWP) and a polarizer (P) allows the switching of the detection polarization state for *x*‐polarization and *y*‐polarization. In the sensor crystal, the polarization of the probe beam is modulated by the THz field via the Pockels effect, whereas the reflected probe beam is captured by the imaging unit. The Wollaston prism (WP) is used to split the probe beam into two beams with orthogonal polarizations, and the two images of the sensor crystal are then projected onto a CCD camera. The THz complex field is extracted using the balanced electro‐optic detection technique. By varying the optical path difference between the THz beam and the probe beam, 64 temporal images are captured at each time delay within a time window of 7 ps. The amplitude and phase information at the different frequencies are extracted by performing Fourier transformations on the temporal signals that occur at each pixel. Finally, the linearly polarized THz fields are detected. The complex amplitude distributions of the LCP and RCP THz waves can be directly calculated from the measured complex amplitudes of the *x*‐ and *y*‐polarized waves using Equation (1). The performances of the used system is measured and are shown in Figure S4 in SI.

**FIGURE 4:**
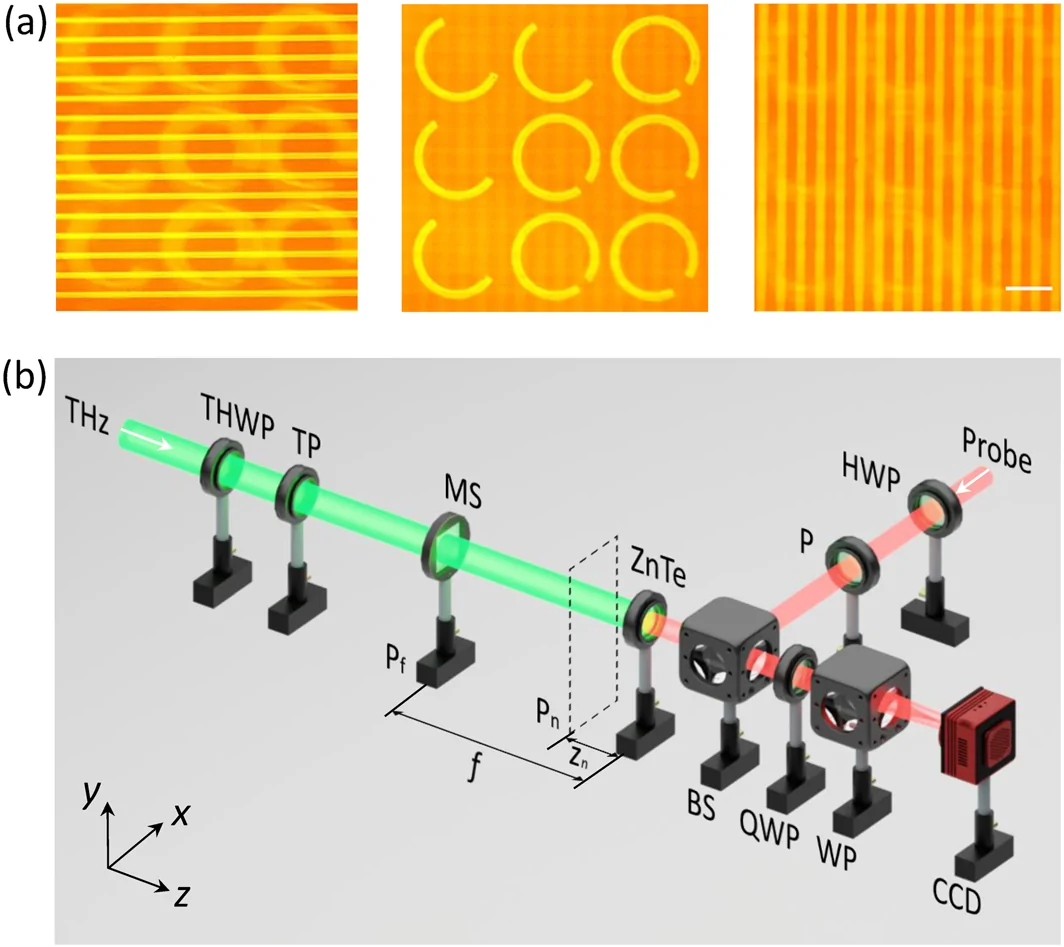
Photograph of the fabricated device and a schematic diagram of the experimental system. (a) Top‐view microscope images of the top, middle, and bottom device layers, respectively. The white bar represents 50 μm. (b) Schematic of the experimental setup. BS, Beam splitter; CCD, Charge‐coupled device; HWP, Half‐wave‐plate, MS, Metasurface, P, Polarizer, QWP, quarter‐wave‐plate, TP, THz Polarizer, TQWP THz quarter‐wave‐plate, WP, Wollaston prism. *P*_f_ and *P*_*n*_ denotes the detection plane of far‐ and near‐field detection, respectively.

We first characterize the polarization‐dependent far‐field response of the fabricated encryption devices. In this experiment, the metasurface samples were illuminated by a 45°‐polarized THz wave and the detection crystal was placed at the far‐field detection plane *P*_f_. The resulting intensity and phase distributions at the focal plane are shown in Figure 5. Figure 5a presents the intensity and phase profiles of the transmitted *x*‐polarized wave for samples #S1, #S2, and #S3. Each image has an area of 9 × 15 mm^2^. The intensity patterns clearly display the digits “6,” “8,” and “9,” respectively. The corresponding phase distributions indicate that the phase of focal spots, exhibit values close to 0, which are consistent with the design. The intensity and phase distributions for the *y*‐polarized wave are shown in Figure 5b. One can see that the intensity patterns shown the same digit shapes with the *x*‐polarization case. The signal‐to‐noise ratio of *y*‐polarized foci is slightly higher. This improvement can be attributed to the larger effective metasurface area contributing to the *y*‐polarized transmission, which results in better focusing performance at the focal plane. The phase values at the focal spots closely match the designed targets of 0, −π/2, and π/2. Figure 5c displays the reconstructed intensity distributions for LCP and RCP waves, which are obtained by coherent superposition of the measured *x*‐ and *y*‐polarized complex amplitudes. For LCP wave, the three samples shown the digits “1,” “0,” and “3,” respectively, whereas RCP wave yields the digits “5,” “2,” and “4.” These results confirm the feasibility of our polarization‐sensitive encryption approach based on independent control of the *x*‐ and *y*‐polarized responses.

**FIGURE 5:**
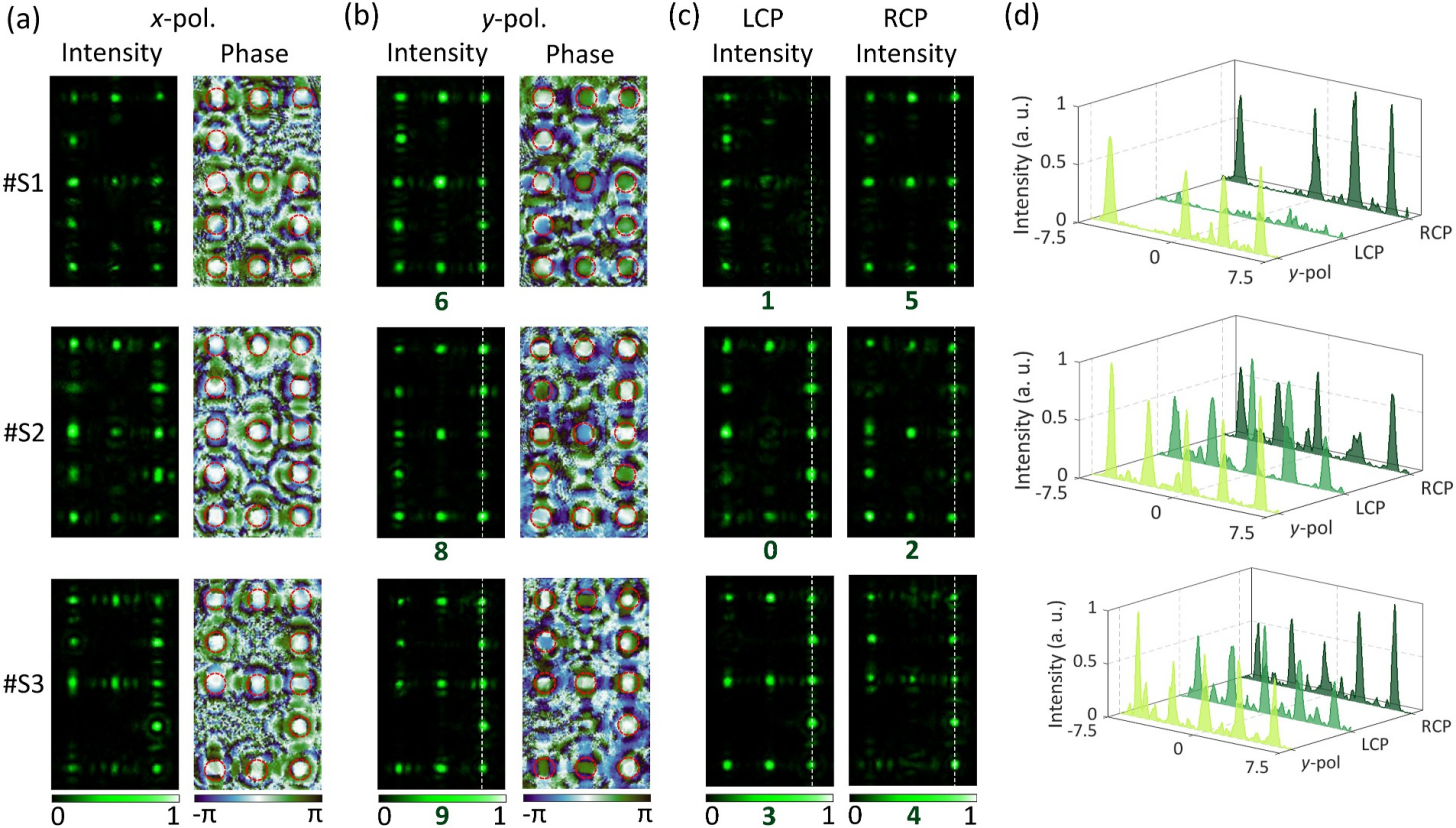
Experimental demonstration of polarization‐sensitive far‐field encryption with the fabricated metasurfaces. (a) Measured intensity (first column) and phase (second column) distributions of the transmitted *x*‐polarized THz wave at the focal plane for samples #S1, #S2, and #S3. Red dashed circles mark the focal spots, whose phases are close to 0. (b) Corresponding intensity and phase distributions for the *y*‐polarized component. (c) Reconstructed intensity patterns for LCP (first column) and RCP (second column) waves. (d) Intensity profiles along the *y*‐axis at *x* = 7.5 mm for the *y*‐polarized, LCP, and RCP distributions shown in panels (b) and (c).

To further evaluate the detail of the focal‐spot arrays, the intensity profiles along the *y*‐axis at the position of *x* = 7.5 mm were extracted for the *y*‐polarized, LCP, and RCP cases, which are shown in Figure 5d. The experimental measured profiles reveal some non‐uniformity in the focal‐spot intensities (See detailed analyses in Part 5 of SI). This may be caused by an imbalance in the total near‐field energy between the *x*‐ and *y*‐polarized components, which can affect the purity of the superposed circular polarizations. Additionally, the minor error in the polarization angle of the incident and detection polarization states could also introduce further background noise. To mitigate this issue, future designs could incorporate simultaneous amplitude and phase modulation to better balance the transmitted energies among different letter‐block regions while preserving the required wavefront shaping functionality [43]. In addition, further optimization of the encoded letter geometries, such as their dimensions and aspect ratios, could help equalize the transmitted energies in the *x*‐ and *y*‐polarized channels, thereby improving the uniformity of the far‐field focal‐spot array and reducing the associated LCP/RCP crosstalk. Despite these imperfections, both the intensity distributions and the line profiles allow clear identification of the presence or absence of focal spots, successfully validating the polarization‐sensitive far‐field encryption functionality of the proposed devices.

We next characterize the intensity distributions of the fabricated encryption devices under near‐field detection, the experimental results are shown in Figure 6. Figure 6a,b display the near‐field intensity profiles of the transmitted *x*‐ and *y*‐polarized THz waves (with samples positioned at *P*_*n*_), respectively, under illumination by a 45°‐polarized THz wave for samples #S1, #S2, and #S3. In the *x*‐polarization channel, the three samples sequentially reveal the words “METASURFACES,” “MULTI‐CHANNEL,” and “IMAGE‐HIDING.” Correspondingly, the *y*‐polarization channel shows the complementary patterns of the same words. Each patterned area measures 9 × 15 mm^2^. These results demonstrate that our designed and fabricated metasurface devices can effectively manipulate the THz information in the near‐field to display intended patterns, thereby providing an experimental foundation for THz near‐field encryption devices. The minimum observed feature width is approximately 500 μm, which is consistent with the designed linewidth of the encoded pattern. This result confirms that the near‐field images produced by the metasurface device are clearly distinguishable and sufficient for presenting the encoded information. Here, the reported ∼500 μm feature width is mainly determined by the intentionally designed linewidth of the encoded letter strokes rather than the intrinsic limit of the THz imaging system. Since the operating wavelength is 400 μm at 0.75 THz, a linewidth slightly larger than the wavelength was chosen to ensure sufficient contrast and reliable near‐field readout quality. In the present system, the spatial resolution of the THz focal‐plane imaging system is ∼32 μm, whereas the metasurface period is 100 μm, which sets the fundamental structural scale for the encoded near‐field pattern. In principle, the near‐field resolution could be further improved by reducing the encoded linewidth toward the metasurface period, although such optimization should also account for the transmitted energy distribution and balance of the x‐ and y‐polarized channels.

**FIGURE 6:**
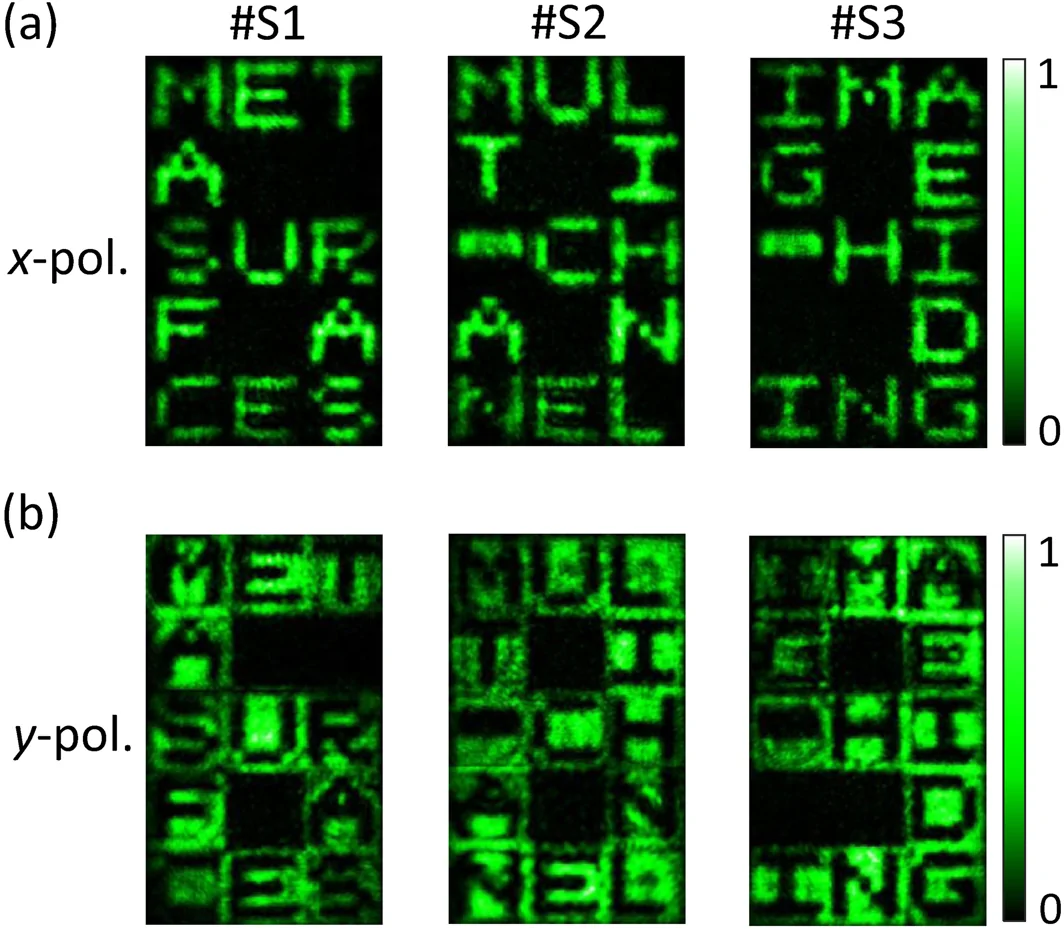
Near‐field characterization of the fabricated device. (a) Measured near‐field intensity distributions of the transmitted *x*‐polarized THz waves for samples #S1, #S2, and #S3 under 45° linearly polarized incidence (detection plane *P*_*n*_). Each intensity distribution has an area of 9 × 15 mm^2^. (b) Corresponding *y*‐polarized near‐field intensity distributions for the same three samples.

Using a tri‐layer metallic metasurface that independently controls the amplitude and phase for *x*‐ and *y*‐polarized waves offers a versatile platform for multichannel polarized information encoding and decoding [41]. It possesses advantage which is the *x*‐unit (*y*‐unit) exhibits nearly zero amplitude transmission for *y*‐polarized wave (*x*‐polarized wave) (see details in Part 1 of SI). This design fundamentally eliminates cross‐talk between the *x*‐ and *y*‐channels. This tri‐layer metasurface also offers the advantage of broadband operation. Although our encryption metasurface design is centered at 0.75 THz, experiments show that it can also perform polarization‐sensitive information encryption function at other frequencies. As shown in Figure S5 in Part 5 of SI, the far‐field measurements at 0.4 THz and 0.9 THz still clearly reveal the polarization‐dependent digital patterns formed by focal‐spot arrays. It should be noted that, since the phase distribution of the metasurface is fixed and will not change along with the working frequency, the corresponding far‐field observation distances (equal to the focal lengths) are 7.5 and 3.3 mm for 0.4 THz and 0.9 THz, respectively. Moreover, near‐field measurements at these two frequencies are shown in Supporting Information S1: Figure S6 in SI, which successfully display the predesigned text patterns. These results indicate that the proposed metasurface encryption device possesses a bandwidth of 0.5 THz. Considering the practical alignment accuracy of the UV lithography process, we further analyzed the influence of representative interlayer displacement and rotational errors on the amplitude and phase responses of the metasurface units, which confirms the fabrication robustness of the present tri‐layer design (see SI, Part 7 and Supporting Information S1: Figure S9). In this work, we demonstrate far‐field encryption using digit patterns composed of focal‐spot arrays, which enables information retrieval but inherently limits the information density. In the future, higher‐capacity encryption devices could be realized by designing metasurfaces that generate Fraunhofer far‐field holograms, thereby achieving high‐resolution holographic image encryption and significantly increasing the information capability of the device.

## 4 Conclusion

In conclusion, we have experimentally demonstrated a dual‐field THz encryption platform based on a single polarization‐sensitive metasurface, which can simultaneously manipulate near‐ and far‐field information through independent control of the amplitude and phase for orthogonally polarized waves. In the near field, the metasurface encodes text patterns that are selectively revealed under specific linear polarization states, whereas in the far field, it generates polarization‐dependent images composed of focal‐spot arrays, enabling distinct digital information to be independently encrypted under LP, LCP, and RCP detection. By fundamentally decoupling the *x*‐ and *y*‐polarized responses, our design overcomes the inherent cross‐talk between different polarized channels. The measured near‐field and far‐field results confirm clear pattern recognition with minimal inter‐channel crosstalk, thereby validating the practicality of this approach for implementing multilevel physical‐layer security. This platform establishes a versatile foundation for developing compact, high‐security THz components applicable to next‐generation communication systems and advanced anticounterfeiting technologies.

## 5 Experimental Section

### 5.1 Numerical Simulation

Commercial software utilizing the finite‐difference time‐domain (FDTD) algorithm is employed to construct the model and simulate the amplitude, phase, and polarization modulations of the metasurface. The simulation employs periodic boundary conditions in the *x*‐ and *y*‐directions, and a perfectly matched layer (PML) in the *z*‐direction to mimic infinite space and minimize reflections. The light source is set as a linearly polarized plane wave spanning a frequency range of 0.1–1.5 THz, which is positioned 5 mm away from the bottom layer of the metasurface. A monitor, placed 5 mm above the top layer of the metasurface, is used to capture the transmitted electric fields components *E*_*x*_, *E*_*y*_, and *E*_*z*_. The size of the computational mesh grid is set to 2 μm in all *x*, *y*, and *z* directions. The dielectric spacer layers consist of PI with a permittivity of *ε* = 2.99. The three metallic layers are set to be perfect electric conductor (PEC).

### 5.2 Sample Fabrication

The designed metasurface devices were fabricated with conventional UV lithography, thermal evaporation, and lift‐off techniques. Regular lithography was firstly carried out, and then lift‐off was performed to obtain the bottom‐layer metal metagrating on a 500‐μm‐thick high‐resistance silicon substrate. The PI was then spin‐coated on the metagrating four times. After each spin‐coating, gradient baking of the sample was done to turn the PI into a solid, and an exact 10‐μm‐thick PI sample was obtained. After four rounds of coating and baking, a 40‐μm‐thick PI spacer was obtained. Then, regular lithography and lift‐off were followed to obtain the metal split‐ring antenna array of the middle layer with highly precise alignment with the bottom layer metagrating. Then, the coating and baking were repeated to obtain another 40‐μm‐thick PI spacer on the metal split‐ring antenna array. Finally, regular lithography and lift‐off were done to obtain the top layer of the metallic metagrating.

### 5.3 Experimental Setup

A THz focal‐plane imaging system was used to characterize the performance of the designed devices. A laser beam with a central wavelength of 800 nm and average power of 2.3 W was divided into two parts, which were used as the pump (2 W) and probe (300 mW) beams for generating and detecting THz waves, respectively. The pump beam impinged on a 1‐mm‐thick 〈110〉 ZnTe crystal to generate the THz beam via optical rectification. The horizontally polarized (*x*‐polarized) THz beam (with a diameter of 20 mm) passed through a THz polarizer to maintain its polarization, then passed through a THz half‐wave plate to generate 45°‐polarized light at a working frequency of 0.75 THz, and then impinged on the structure. The scattered THz beam was detected using another 〈110〉 ZnTe crystal. The THz complex field was extracted using balanced electro‐optic detection. The detected polarization state can be tuned into *x*‐ or *y*‐polarization state by changing the polarization state of the probe beam.

## Author Contributions

**Yixuan He:** writing – original draft, visualization, data curation. **Huan Zhao:** supervision, project administration, visualization, writing – original draft, writing – review and editing, conceptualization, investigation. **Tong Nan:** software, writing – original draft. **Xinke Wang:** methodology, validation, writing – original draft, formal analysis. **Yan Zhang:** writing – review and editing, resources, supervision, project administration, funding acquisition.

## Conflicts of Interest

The authors declare no conflicts of interest.

## Supporting information


Supporting Information S1

## Data Availability

The data that support the findings of this study are available from the corresponding author upon reasonable request.
