# Supplementary material for: Terahertz Encryption With a Polarization‐Sensitive Metasurface
Source: Nanophotonics. 2026 May 6;15(9):e70123. doi: 10.1002/nap2.70123 (PMC13642153; doi:10.1002/nap2.70123)
Supplement: Supplementary file 1 [file NAP2-15-e70123-s001.docx]

**Supplementary Information for**

**Terahertz Encryption with a Polarization-sensitive Metasurface**

Yixuan He, Huan Zhao*, Tong Nan, Xinke Wang, and Yan Zhang

Y. He, T. Nan, X. Wang, Y. Zhang

Department of Physics

Capital Normal University

Beijing, 100048, China

H. Zhao

School of Optics and Photonics

Beijing Institute of Technology

Beijing, 100081, China

E-mail: zhaohuan@bit.edu.cn

**1. Numerical simulation and experimental characterization of metasurface units**

Commercial software *FDTD Solutions* which utilizing the Finite-Difference Time-Domain (FDTD) algorithm is employed to construct the model and simulate the amplitude and phase modulations of the metasurface. The simulated model employs periodic boundary conditions in the *x*- and *y*-directions, and a perfectly matched layer (PML) in the *z*-direction to simulate the infinite space and minimize reflections. The light source is set as a linearly polarized plane wave spanning a frequency range of 0.1-1.5 THz, which is positioned 5 mm away from the top layer of the metasurface. A monitor, placed 5 mm away from the bottom layer of the metasurface, is used to capture the transmitted electric fields components *E_x_* and *E_y_*. The size of the computational mesh grid is set to 2 μm in all *x*, *y*, and *z* directions. The dielectric spacer layers consist of PI with a permittivity of *ε* = 2.99. The material of the three metallic layers is set to be perfect electric conductor (PEC).

To experimentally validate the Jones-matrix design of the metasurface units, we further characterized the transmission amplitudes of the four Jones-matrix elements, $t_{xx}$, $t_{xy}$, $t_{yx}$, and $t_{yy}$, for representative *y*-unit #1 and *x*-unit #1 using the THz focal-plane imaging system. Here, the first and second subscripts of $t$ denote the incident and transmitted polarization states, respectively. The measured results were compared with the corresponding numerical simulations, and the results are shown in **Figure S1**. For *y*-unit #1, the cross-polarized transmission component $t_{xy}$ is dominant over the 0.4–0.9 THz range, while the other components remain strongly suppressed. Similarly, for *x*-unit #1, the $t_{yx}$ component is dominant, whereas the undesired transmission components stay at low levels. The measured spectra agree well with the simulated results, confirming that the designed tri-layer metasurface units function as efficient cross-polarization converters with effectively suppressed unwanted transmission channels.

 **Figure S1.** Simulated and measured transmitted amplitude spectra of the Jones-matrix elements for representative *y*-unit #1 and *x*-unit #1. a) Simulated and measured $t_{xx}$, $t_{xy}$, $t_{yx}$, and $t_{yy}$ of *y*-unit #1. b) Simulated and measured $t_{xx}$, $t_{xy}$, $t_{yx}$, and $t_{yy}$ of *x*-unit #1. The first and second subscripts of $t$ denote the incident and transmitted polarization states, respectively.

We have selected 16 *y*-unit metasurfaces and 16 *x*-unit metasurfaces to modulate the transmitted *y*- and *x*-polarized THz wave. The detailed data of these C-shaped antennas are shown in **Figure S2**. All these metasurface share the same gratings and space layers but with different C-shaped antennas. All these C-shaped antennas have the same outer and inner radius but with different opening angle and symmetric angle.

**Figure S2.** The detailed data of the C-shaped antennas using in all the selected metasurfaces. *α* denotes the opening angle of the antenna. Symmetric angle of the antenna is defined as the angle between the symmetric axis of the antenna and *x*-axis.

**2. Fresnel diffraction algorithm for far-field numerical simulation**

The Fresnel Diffraction (FD) was used to calculate the far-field intensity and phase distribution of the metasurface. The FD formula can be expressed as:

$U_{out}\left( x_{1},y_{1} \right)=\frac{e^{jkz}}{j\lambda z}\iint U_{ms}\left( x_{0},y_{0} \right)\cdot e^{\frac{jk}{2z}\left[ \left( x_{0}{-x}_{1} \right)^{2}+\left( y_{0}{-y}_{1} \right)^{2} \right]}dx_{0}dy_{0},$	(S1)

where *U_obj_* (*x*_1_, *y*_1_) and *U_ms_* (*x*_0_, *y*_0_) denotes the image plane and the metasurface plane, respectively. (*x*_1_, *y*_1_) and (*x*_0_, *y*_0_) are the coordinates of the image plane and metasurface plane respectively, *λ* is the working wavelength which is 400 μm (0.75 THz), and *k* is the corresponding wave vector. *z* is the distance between the far-field detected plane P_f_ and the metasurface device, which is equals to the focal length *f* = 4 mm. The pixel numbers of the *U_out_* and *U_ms_* are 300×180 and 150×90, respectively. The pixel size of the *U_out_* and *U_ms_* are 50 and 100 μm, respectively.

**3.** **Fabrication flow of the proposed metasurface**

The fabrication flow for the proposed metasurface is shown in **Figure S3**. The designed metasurface devices were fabricated with conventional UV lithography, thermal evaporation, and lift-off techniques. The fabrication workflow is illustrated in Fig. 9. Regular lithography was firstly carried out, and then lift-off was performed to obtain the bottom-layer metal metagrating on a 500-μm-thick high-resistance silicon substrate. The PI was then spin-coated on the metagrating four times. After each spin-coating, gradient baking of the sample was done to turn the PI into a solid, and an exact 10-μm-thick PI sample was obtained. After four rounds of coating and baking, a 40-μm-thick PI spacer was obtained. Then, regular lithography and lift-off were followed to obtain the metal C-shaped antenna array of the middle layer with highly precise alignment with the bottom layer metagrating. Then, the coating and baking were repeated to obtain another 40-μm-thick PI spacer on the metal split-ring antenna array. Finally, regular lithography and lift-off were done to obtain the top layer of the metallic metagrating.

**Figure S3**. The schematic diagram of fabrication workflow of the tri-layer metallic metasurface.

**4.** **Characteristics of the THz focal-plane imaging system**

**Figure S4a** and **4b** show the time-domain signal and frequency-domain spectrum of the THz wave generated for the THz focal-plane imaging system, respectively. The duration of the THz pulse is about 2 ps, while the frequency range is approximately 2 THz. The amplitude distribution of the incident THz wave at 0.75 THz is shown in **Figure S4c**. The diameter of the spot is approximately 16 mm. The amplitude along the white dashed line in **Figure S4c** is extracted and shown in **Figure S4d**. The amplitude distribution of the THz beam shown Gaussian-like shape, which affects the quality of the decoded information.

**Figure S4**. Characteristics of the THz focal imaging system. a) Measured time-domain signal. b) Measured frequency-domain spectrum. c) Measured amplitude distribution of the illuminating wave at 0.75 THz. d) Amplitude distribution extracted along the white dashed line in c).

**5. Quantitative evaluation of far-field focal-spot non-uniformity**

To quantitatively evaluate the non-uniformity of the far-field focal-spot arrays, both the metasurface and the generated near-field/far-field information was divided into $5\times3$ regions, corresponding to 15 letter-block or focal-spot regions. Based on this partition, the local energy associated with each focal spot was evaluated and compared across the full array. The spot-to-spot uniformity was quantified by the coefficient of variation (CV), defined as:

$\mathrm{CV}=\frac{\sigma}{\mu},$	(S2)

where $\sigma$ and $\mu$ denote the standard deviation and mean value, respectively, of the local energies of all selected focal spots. Specifically, for a set of $N$ focal spots with local energies $\left\{ E_{i}{\}}_{i=1}^{N} \right.$,

$\mu=\frac{1}{N}\sum_{i=1}^{N} E_{i},\sigma=\sqrt{\frac{1}{N-1}\sum_{i=1}^{N} (E_{i}-\mu)^{2}}.$	(S3)

Here, $E_{i}$ is defined as the integrated energy within a region centered at the corresponding focal spot, with a spatial extent equal to three times the full width at half maximum (FWHM) of that spot. A smaller CV indicates a more uniform focal-spot array. The corresponding CV values obtained from the simulated and experimental focal-spot intensities are summarized in **Table S1**.

**Table S1.** Calculated coefficient of variation of far-field focal-spot array

| Sample | | x-polarized | y-polarized | LCP | RCP |
| --- | --- | --- | --- | --- | --- |
| #S1 | Sim. | 0.16 | 0.09 | 0.17 | 0.19 |
|  | Exp. | 0.20 | 0.18 | 0.21 | 0.23 |
| #S2 | Sim. | 0.15 | 0.11 | 0.16 | 0.18 |
|  | Exp. | 0.21 | 0.19 | 0.23 | 0.24 |
| #S3 | Sim. | 0.16 | 0.13 | 0.17 | 0.19 |
|  | Exp. | 0.19 | 0.17 | 0.20 | 0.25 |

To further analyze the origin of the observed non-uniformity, sample #S1 was taken as a representative example. The near-field *x*- and *y*-polarized energies associated with each of the 15 metasurface letter-block regions were numerically examined, together with the corresponding focal-spot intensities in the far field. Because the near-field *x*- and *y*-polarized information is encoded as complementary patterns of the word “METASURFACES”, the total near-field energies in different letter-block regions are inherently non-uniform for the two polarization channels. The normalized near-field energy distributions of the *x*- and *y*-polarized channels in the 15 letter-block regions of sample #S1 are shown in **Figure S5a** and **S5b**, respectively, where each map is normalized to the maximum transmitted energy within the corresponding polarization channel. The corresponding simulated far-field focal-spot intensity distributions of the *x*- and *y*-polarized channels in the 15 regions are shown in **Figure S5c** and **S5d**. The relative intensity distribution among the output focal spots in each polarization channel is correlated with the relative near-field energy distribution of the corresponding input letter blocks. This result indicates that the non-uniform intensity distribution among different letter regions, originating from the near-field letter-pattern modulation, is a major contributor to the non-uniformity of the far-field focal-spot array. In addition, when the *x*- and *y*-polarized components are further combined to form the LCP and RCP channels, this imbalance also affects the inter-channel crosstalk between the LCP and RCP outputs, and therefore influences the corresponding signal-to-noise ratio.

**Figure S5.** Representative analysis of the physical origin of focal-spot non-uniformity for sample #S1. a) and c) Normalized near-field energy distribution of the *x*- and *y*-polarized channels in the 15 letter-block regions of sample #S1. b) and d) Corresponding simulated far-field focal-spot intensity distributions of the x- and y-polarized channels in the 15 regions.

**6. Characteristics of broadband** **property of the metasurface device**

We experimentally characterized the broadband performance of the metasurface device. Leveraging the broadband capability of the measurement system and the high-efficiency-cross-polarization conversion of the metasurface in a wide spectrum, the near- and far-field encryption performance at 0.4 THz and 0.9 THz are demonstrated experimentally. **Figure S6** shows the polarization-dependent far-field encryption results for samples #S1, #S2, and #S3 at these two frequencies. One can see in all cases, the designed digital patterns composed of focal spot arrays are generated. It should be noted that, because the phase profile of the metasurface is fixed and does not change with frequency changing, the effective focal lengths shift to approximately 7.5 mm at 0.4 THz and 3.3 mm at 0.9 THz. **Figure S7** shown the corresponding near-field measurement results. Under both *x*- and *y*-polarized illumination, the correct word patterns and their complementary inverses are successfully displayed at both frequencies. These results confirm that the tri-layer metallic metasurface encryption device can operate over a broad band, with a bandwidth of 0.5 THz.

**Figure S6**. Experimental characteristics of the broadband encryption property of the metasurface in the far-field. Measured far-field intensity distributions at a) 0.4 THz and b) 0.9 THz.

**Figure S7**. Experimental characteristics of the broadband encryption property of the metasurface in the near-field. Measured near-field intensity distributions at a) 0.4 THz and b) 0.9 THz.

To quantitatively evaluate the broadband far-field encryption performance of the proposed metasurface, we analyzed the signal-to-noise ratio (SNR), the inter-channel crosstalk between the LCP and RCP channels, and the pattern resolution at different frequencies based on the experimental results. Specifically, the SNR of the far-field focal-spot array was defined as the ratio between the average intensity of the desired focal-spot regions and the average background intensity of the regions where no focal spot was designed to appear. For each sample, the far-field pattern was divided into 15 predefined regions corresponding to the 5 × 3 focal-spot array. Among them, $N$ regions contain the intended focal spots for a given decoded pattern, while the remaining $15-N$ regions correspond to non-focal regions. For each desired focal spot, the effective focal region was defined as a local area centered at the focal spot with a spatial extent equal to three times its full width at half maximum (FWHM). The SNR is then defined as

$\mathrm{SNR}=\frac{\frac{1}{N}\sum_{i=1}^{N} \bar{I}_{i}^{\mathrm{sig}}}{\frac{1}{15-N}\sum_{j=1}^{15-N} I_{j}^{\mathrm{bg}}}$	(S4)

where $\bar{I}_{i}^{\mathrm{sig}}$denotes the average intensity within the effective focal region of the $i$-th desired focal spot, and $I_{j}^{\mathrm{bg}}$denotes the average intensity within the $j$-th non-focal region. A larger SNR indicates better distinguishability of the encoded focal-spot array from the background. For each frequency, the SNR values were first calculated for all four polarization channels of the three samples, and the final SNR at that frequency was obtained by averaging these 12 values.

Next, we quantified the inter-channel crosstalk between the far-field LCP and RCP experimental results. For each sample, the far-field pattern was divided into 15 predefined regions corresponding to the 5 × 3 focal-spot array. Among them, $N$ regions were selected, where a focal spot was designed to appear in only one circular-polarization channel, namely LCP or RCP, but not in both simultaneously. For the $k$-th selected region, the local crosstalk was defined as the ratio between the average intensity in the undesired channel and the average intensity in the desired channel. The overall crosstalk between the LCP and RCP channels is then defined as

$\mathrm{CT}=\frac{1}{N}\sum_{k=1}^{N} \frac{J_{k}^{\mathrm{leak}}}{J_{k}^{\mathrm{sig}}},$	(S5)

where $J_{k}^{\mathrm{sig}}$denotes the average intensity within the $k$-th region in the polarization channel where a focal spot is intentionally designed to appear, and $J_{k}^{\mathrm{leak}}$denotes the average intensity within the same region measured in the opposite polarization channel, where no focal spot is expected. A smaller $\mathrm{CT}$ indicates weaker inter-channel leakage, and $\mathrm{CT}=0$ corresponds to the ideal case of zero crosstalk between the LCP and RCP channels. For each frequency, the crosstalk values were first calculated separately for the three samples, and the final crosstalk at that frequency was obtained by averaging these three values.

Finally, the pattern resolution of the far-field focal-spot array was characterized by the full width at half maximum (FWHM) of the desired focal spots. For each frequency, the FWHM of each intended focal spot was measured from the corresponding far-field intensity distribution, and the average FWHM over all desired focal spots was used as the resolution-related metric for that frequency. A smaller average FWHM corresponds to a sharper focal-spot array and better separability of the encoded pattern. The results are summarized in **Figure S8**. As shown in **Figure S8a**, the SNR exhibits an overall increase from 13.7 at 0.4 THz to a maximum value of 18.5 at 0.75 THz, followed by a slight decrease at higher frequencies. In contrast, the crosstalk remains low over the full frequency range, varying between 0.06 and 0.13, and reaches its minimum value at 0.75 THz. This indicates that the far-field decoding performance is achieved well near the design frequency. Away from 0.75 THz, although the SNR decreases and the crosstalk slightly increases, the encoded focal-spot patterns remain clearly distinguishable. This behavior is mainly attributed to the fact that the metasurface is optimized at 0.75 THz. When the operating frequency moves away from the design frequency, the phase modulation of the metasurface units gradually deviates from the target values, which introduces a slight phase mismatch in the designed far-field interference. As a result, the focal-spot contrast is reduced and the residual leakage between the LCP and RCP channels becomes more noticeable, leading to a relatively higher crosstalk. **Figure S8b** shows the average full width at half maximum (FWHM) of the desired focal spots as a resolution-related metric. The average FWHM decreases from 848 μm at 0.4 THz to 402 μm at 0.9 THz. This trend is mainly related to the decrease in wavelength at higher frequencies. For a given aperture, a shorter wavelength generally leads to a more tightly confined diffraction-limited focal spot in the far field, and therefore a smaller FWHM.

**Figure S8.** Quantitative evaluation of the broadband far-field encryption performance of the metasurface. a) Frequency-dependent SNR and inter-channel crosstalk between the LCP and RCP channels. b) Frequency-dependent average FWHM of the desired focal spots.

**7. Tolerance analysis of fabrication-induced interlayer alignment errors**

To evaluate the fabrication robustness of the proposed tri-layer metallic metasurface, we further analyzed the influence of practical interlayer alignment errors on the modulation performance of the metasurface units. In the UV lithography process used in this work, cross-shaped alignment marks together with their inverse structures were designed on the four sides of the three masks to assist the layer-by-layer alignment. Since the intermediate spacer layers are made of PI, which is transparent in the visible range, the relative positions between adjacent layers can be directly monitored by a microscope coupled with a CCD system during alignment. Based on our fabrication assessment, the relative lateral displacement between adjacent layers is below 5 μm, and the interlayer rotational error is within 2°.

Based on these practically relevant error ranges, we first simulated the transmitted amplitude spectra of representative *y*-unit #1 under a 5 μm lateral shift of the top grating and under a rotational error of ±1°, and the results are shown in **Figure S9a**. It can be seen that a 5 μm lateral displacement introduces only a minor change in the transmitted amplitude. This is because the metasurface period used in this work is 100 μm, while the period and linewidth of the top and bottom gratings are 20 μm and 10 μm, respectively. Therefore, a 5 μm relative shift does not significantly alter the overall polarization-selective transmission behavior of the unit. In contrast, a rotational error of the grating leads to a slight decrease in the desired cross-polarized transmission amplitude, because the gratings function as polarization filters in the tri-layer metasurface. Nevertheless, the influence of a 2° rotational deviation remains within an acceptable range.

We further analyzed the phase modulation robustness of the metasurface units by simulating *y*-unit #1–#16 under the same 5 μm lateral shift and 2° rotational error. The corresponding phase modulation of the transmitted *y*-polarized wave are shown in Figure S9b. It is found that the phase modulation remains relatively robust against these practical alignment perturbations. This is mainly because the phase modulation is primarily determined by the opening angle of the C-shaped antenna in the middle layer. Therefore, within the experimentally relevant alignment-error range, the influence of fabrication-induced interlayer misalignment on the far-field spot crosstalk is expected to be very limited.

These results indicate that the proposed tri-layer metallic metasurface possesses sufficient tolerance to practical fabrication errors achievable in our UV lithography process, which supports the feasibility of the present device for experimental implementation.

**Figure S9.** Tolerance analysis of fabrication-induced interlayer alignment errors. a) Simulated transmitted amplitude spectra of representative *y*-unit #1 under ideal alignment, a 5 μm lateral shift of the top grating, and a rotational error of 2°. b) Simulated phase modulation of *y*-unit #1–#16 for the transmitted *y*-polarized wave under ideal alignment, a 5 μm lateral shift, and a rotational error of 2°.
